# Nurse Prescribing-Readiness of Polish Nurses to Take on New Competencies—A Cross-Sectional Study

**DOI:** 10.3390/healthcare7040151

**Published:** 2019-11-21

**Authors:** Anna Bartosiewicz, Andrzej Różański

**Affiliations:** 1 Institute of Health Sciences, Medical College of Rzeszow University, 35-959 Rzeszow, Poland; 2 Department of Labour Pedagogy and Andragogy, Faculty of Pedagogy and Psychology, Maria Curie-Sklodowska University, 20-004 Lublin, Poland; rozanski@umcs.pl

**Keywords:** drugs, nurse, prescribing, readiness

## Abstract

From January 2016, nurses in Poland received new competencies for prescribing. The work is the first in Poland to elaborate on the subject of nurses’ readiness to learn and develop in the context of new nursing rights regarding autonomous prescription of medication and continuation of the prescription for medication. The aim of the study is to analyze the readiness of Polish nurses to learn and develop in the context of new competencies to write prescriptions. The research was conducted among 756 nurses. The standardized questionnaire (Readiness of Employees for Learning and Development) was used. For all subscales of readiness for learning and development, average scores prevailed. The readiness to write prescriptions was significantly related to the level of openness to changes in the work environment (A1 scale), self-evaluation of past educational development (C5 scale) and educational and professional goals alignment- employee and company (scale D2) and increasing the readiness of nurses to practice all of the aforementioned agents, in particular medical devices. The readiness of nurses to learn and develop at all levels of the subscales was on an average level. Younger nurses, with a shorter seniority, having higher education and additional qualifications had a higher readiness to prescribe medications and write prescriptions. The higher readiness for learning and development was matched by a greater readiness to prescribe. The results obtained can be used to plan training and courses, as well as to create special pro-development programs, which may increase the nurses’ involvement in personal and professional development.

## 1. Introduction

The development of nursing and the taking over of new functions by nurses have made nursing in many countries a more complex profession, allowing for high-quality patient care [1,2].

The introduction on 1 January 2016 of new entitlements for Polish nurses authorizing the prescription of medicines, foodstuffs for particular nutritional purposes and medical devices seems to be a response to the changes taking place in this profession [3]. According to the position of the International Nurses’ Council, the implementation of new nursing competencies will improve the standards of patient care and increase the availability of medical services, help to use nurses’ skills and improve their professional status [2]. Nurse prescribing is a novelty in Poland, which raises a lot of controversy and doubt, but it is a great breakthrough in Polish nursing and the first step to introducing the role of an advanced practice nurse. The new rights allow for more effective work with the patient, while imposing additional responsibility on the nurses and the need to undertake training to supplement knowledge in the field [4].

The first country that gave nurses permission to prescribe medicines was the United States in the 1970s. Other countries like Australia, Botswana, Canada, Great Britain, Ireland, the Netherlands, New Zealand, Spain and Sweden, also have wide experience in this field [2,5,6,7,8,9,10].

The dynamic development in a medicine specific work environment places high demands on health care workers [11,12]. The ongoing process of knowledge update and postgraduate education is particularly important [12,13]. The continuous development of medical science, the introduction of new diagnostic methods and treatment with the use of specialized equipment as well as the development of nursing makes the knowledge acquired during studies insufficient. In connection with the above, practicing the profession of a nurse in accordance with the indications of up-to-date knowledge obliges them to raise their professional qualifications and acquire new competencies. It creates an idea that can be named “Fashion for knowledge” [14,15,16].

In many countries, the issue of in-service training for nurses is an important element of lifelong learning (CPD—Continuum Professional Development) [17,18]. Readiness and aspiration to continue development are among the main features attributed to the role of 21st century nurses, especially in the countries where nurses are an important and numerous professional group in the health care system as in Great Britain, Ireland, Netherlands, or Norway [1,2,19,20,21]. The current education of nurses in Poland takes place at the academic level of pre-graduate education: the first degree lasts three years, which ends with obtaining a bachelor’s degree. Second degree studies last two years to obtain a master’s degree in nursing [22]. Nurses can also improve their qualifications in post-graduate education. According to the Polish legislation there are four types of postgraduate education for nurses:

- Specialization training called “specialization”, enabling qualifications in a specific field and obtaining the title of specialist in this field. This training ends with a state exam, which is conducted by an examination committee appointed by the Minister of Health.
- Qualification course- enabling acquisition of the qualifications needed in a given field of nursing to provide specific health services, falling within the scope of a given field of nursing or a field applicable in health care.
- Specialist course enabling the nurses to obtain the qualifications necessary to perform specific professional activities while providing nursing, preventive, diagnostic, therapeutic or rehabilitation services.
- Further training courses aimed at deepening and updating the knowledge and skills of professional nurses and midwives [23].

New competencies for Polish nurses should be considered in two aspects: as an independent decision regarding the assignment of a drug to a patient resulting from knowledge and physical examination and as a continuation of treatment previously ordered by a doctor. Nurses with a master’s degree in nursing can independently order specific medications, while nurses after completing their first-cycle studies can only write prescriptions for medication necessary to continue treatment [3]. Nurses who completed their studies before 2016 are required to complete specialist courses preparing them for writing prescriptions. This situation forced the necessity of changes in nursing education standards. Hence, students who started their studies from the 2016/2017 academic year participate in these courses, because the study program is significantly expanded in this topic. Extended education includes additional effects on knowledge and skills in clinical pharmacology and physical examination, taking into account the patient’s clinical condition [24]. The exact list of medicines that may be prescribed by nurses in Poland and all procedures related to the nursing prescription is regulated by the Regulation of the Health Minister. The list contains 16 groups of medicines containing 31 active substances approved for discharge by nurses. These are, among others: antispasmodics, anticholinergics, antiemetics, topical and gynecological anti-infectives, treatments for anemia, nonspecific antibacterial agents, topical anesthetics, analgesics, anxiolytics, antiparasitics, antiasthmatic drugs, and remedies for migraine [25]. This is not much, especially when compared to other countries such as the USA, Sweden, or Ireland, where nurses have full autonomy in prescribing medicines for their patients [5,7,8,26,27].

The changes in the education standards of the new nursing staff and specialist courses on “ordinance of medicines and writing prescriptions” allow Polish nurses to supplement their knowledge and prepare for new competencies. However, due to legislation in Poland, giving nurses new competencies does not oblige them to take on the new tasks, but only gives them the opportunity [28]. To date, new competencies are not related to an increase in salary or professional promotion but are very responsible tasks. Here, self-determination and desire of the respondents is needed to demonstrate willingness to learn and take on new tasks, although there are no visible benefits, except of course personal satisfaction. It will depend on the decision of the nurses themselves whether they will show willingness to learn and thus benefit from the rights they have been granted [29].

The aim of the study is to analyze the readiness of Polish nurses to learn and develop in the context of new tasks. New competencies are considered in two aspects: as an independent decision regarding the assignment of a drug to a patient resulting from knowledge and physical examination and as a continuation of treatment previously ordered by a doctor.

## 2. Materials and Methods

### 2.1. Ethics Approval and Consent to Participate

The research project was carried out in accordance with the Helsinki Declaration. The study was approved by Bioethics Committee at the University (resolution no. 12/12/2015).

The study was conducted in 2016, among Primary Health Care nurses in the South -Eastern part of Poland. The place where the tests were carried out were chosen randomly at 13 treatment entities, this means Primary Health Care entities. The following criteria for the selection of the study participants were used: nurses with at least one year of professional experience, working in Primary Health Care, and who agreed to participate in the study. Nurses not having one year of work experience, nurses working in hospitals and other medical facilities, and nurses who did not agree to participate in the study were excluded from the study.

The research method used in this work was a diagnostic survey carried out using a questionnaire technique. The author’s survey questionnaire was built based on a five-point Likert scale and contained questions about the opinions and knowledge regarding the prescribing and sociodemographic data of the respondents. In order to examine readiness to prescribe, two components have been distinguished, which sum up 69.2% of the variance in total (the first explains 48.7% of the variance and the other 20.5% of the variance—together they give a satisfactory result of almost 70% of the explained variance). In the first component we have foodstuffs for nutritional use, medical devices and medicines ordered by a doctor, and in the second component, powerful drugs, intoxicants, psychotropic drugs, and antibiotics.

A standardized questionnaire Readiness of Employees for Learning and Development (RELD) was used to assess readiness for learning and development among the studied group of nurses. The author of this questionnaire is Różański dealing with research on readiness for learning and development among adults with their studied profession [30]. This questionnaire was used to assess the pro-development readiness of employees among employees of companies in the medical services industry [30,31,32].

This tool, thanks to the five-point Likert scale, enables the measuring of readiness for learning and development in the workplace. The scale system allows identification of the so-called predisposition of general readiness to learn and develop, and identification of the “predisposition of general readiness to learn and develop”, and identification of the “professional readiness for learning and development” [30].

The RELD questionnaire contains 48 statements divided into 6 theoretical subscales corresponding to the areas studied.

The first three subscales (A1, B3, C5) were concern in the group of named predispositions “general to development and learning”:

- The A1 scale is referred to as the level of openness to changes in environment: This included the individual’s self-knowledge described by wording: I have many interests; I am interested in new ideas for solving emerging problems; it makes me happy to learn new things; I do not care about my comprehensive non-professional intellectual development; I have innate intuition, thanks to which I achieve a lot; I have enough potential to take on completely new challenges; regardless of the situation at work, I try to constantly improve my professional qualifications; I try to draw conclusions from any situation that would help in achieving my future goals; I actively look for opportunities to expand my knowledge and skills; I would like to have an impact on the subject of training organized in my company; I am happy to report my training needs to my superiors; it is good that people have to learn all their lives.
- B3 scale refers level of professional mobility awareness: This allowed individuals to comment on the perception of their professional opportunities in a macro-environment: A difficult situation on the labor market significantly limits my job opportunities; people of my age have little chance of satisfactory work; gender plays a significant role in developing professional careers; men have a better chance of finding an interesting job than women.
- C5 scale named self-evaluation of past educational development: This allowed individuals to respond to their own educational experiences in the context of achieved results. They focused on the following statements: I am well-educated; I have made full use of my own professional development opportunities; I am satisfied with my education; I do not assess my current educational/self-education activity very much; education is a significant value in my life; with learning in the past, I usually achieved very good results/assessments; being in the role of a student, I always apply to learning to achieve satisfying results.

The next three subscales (D2, E4, F6) were focused on the aspects of the so-named “professional readiness for learning and development”:

- D2 scale refers educational and professional goals alignment (employee and company): This enabled assessment in the following areas: Whether the current job fully matches my potential; the employer is interested in my professional development; in connection with the work I am required to improve my qualifications; the employer supports me in my professional development concept; without the help of an employer, I would rather not invest myself in my own development; the training policy in the company suits me perfectly; in case of problems I know who to ask for help in the company; the employer is interested in my being educated not only in terms of work; the industry in which I work has a future.
- E4 scale named inner need of an employee for the professional information: Statements included in this subscale were: Knowledge and skills that I have are perfectly adequate for my professional needs; in my situation, learning things that do not bring professional benefits do not make sense; I usually learn in situations when it is related to my current or future professional work.
- F6 scale refers readiness as effectiveness of on-the-job training: The points of the subscales contained the following statements: Training in the company is generally a waste of time; I feel compelled to participate in training I am not interested in; due to the workload I have too little time for pro-development activities; I get the impression that I take part in too many training sessions Respondents provided answers in the range from 1 to 5; 1 corresponds to “fully disagree” and 5 to “fully agree” [30,31].

The Cronbach’s alpha reliability index for the entire scale was 0.674 [30].

To check the correctness of completed questionnaires in terms of understanding a pilot study was conducted among 34 Primary Health Care nurses. The reliability of the RELD questionnaire subsystem fluctuated within the Cronbach alpha range of 0.284 (E4 scale) to 0.926 (A1 scale). Similarly, the consistency of individual questions building the scale was satisfactory, because most questions correlated positively with each other, and only in a few cases was the correlation weak negative. Similarly, the correlations of particular subscale items with the general results were mostly strongly positive and confirm a good understanding of the tool.

The full research study was conducted among 756 Primary Health Nurses after obtaining approval from the managers of these facilities. The purpose of the study was clarified at meetings. The respondents received oral information about the study and then written information about its purpose and its voluntary nature. The respondents were assured that their consent or refusal to participate would not affect their continued employment in a given health care institution. To ensure data confidentiality, the questionnaires were marked with numbers and placed in sealed envelopes, they were then handed to the respondents directly during face to face meetings. Correctly completed questionnaires were equivalent to the consent of the participants to participate in the study. A total of 1320 questionnaires were distributed, of which 800—i.e., 60%—were collected back. After verifying all the questionnaires, 44 questionnaires were rejected due to the incompleteness of the answers provided. Finally, the data from the 756 questionnaires were plotted on the sheet and analyzed statistically.

### 2.2. Statistical Analysis

The estimation method and the following statistical methods were used:

- To present the data the methods of descriptive statistics were used: the arithmetic mean (M), the value of which determines the average level of a given variable and the standard deviation (SD), the statistical measure of the results spreading around the expected value were used in order to present statistical data.
- The verification of differences between variables measured on the qualitative scale was made using independence test × 2, taking into account in tables 2 × 2, the Yates continuity correction and the Fisher exact test results.
- Differences between quantitative variables were tested using the Mann–Whitney test and Kruskal–Wallis test for calculating the Spearman rho correlation coefficient.
- The calculations were carried out with the IBM SPSS Statistics 20 program.
- The level of significance was *p* < 0.05.

## 3. Results

### 3.1. Characteristics of the Study Group

The average age of the respondents was 47.76 ± 9.65 years and ranged from 22 to 69 years. Over half of the respondents, 411 (54.4%), had worked in the profession of a nurse over 20 years. More than every fourth person, 159 (21.0%), had been employed from 16 to 20 years. Less numerous groups of nurses had been employed in the profession: for 1–5 years, 47 (6.2%); from 6–10 years, 68 (9.0%); or from 11–15 years, 71 (9.4%).

Almost half of the respondents, 343 (45.4%), were nurses with secondary nursing education. The second group in terms of numbers were nurses with the professional title of bachelor of nursing- higher vocational education, 157 (20.8%); and the third one, people with higher education, 104 (13.8%). A nursing education and specialization in nursing was shown by 73 (9.7%) people, while the first degree and nursing specialties were held by 38 (5.0%) nurses. Only 41 (5.4%) people had a second-level Master’s degree and a specialization in nursing.

The most frequently mentioned health care facility in which nurses had basic employment were public health care institutions, full-employment contract, 644 (85.2%). In non-public healthcare institutions, 112 (14.8%) of the respondents were contract workers. Just over half of nurses, 404 (53.4%) had a specialist course, 345 (45.6%) had a qualification course in the field of nursing, almost every third respondent completed a training course, 247 (32.7%). There were 141 (18.7%) people specializing in nursing, and 59 (7.8%) nurses had other forms of postgraduate qualification (post-graduate studies).

### 3.2. Results

For all the levels of readiness for learning and development, average scores predominated. The results should be analyzed on a scale from 1 to 5 points. The most common level was the level of educational and professional goals alignment (employee and company) (*N* = 591, i.e., 78.2%) and the level of effectiveness of on-the-job training (*N* = 580, i.e., 76.7%). In the smallest degree, the average results were reached on the scale of inner need of an employee for the professional information (*N* = 375, i.e., 49.6%), where a significant part of the respondents (*N* = 345, i.e., 46.8%) obtained low results Table 1.

Readiness to ordain drugs/agents was significantly associated with the level of openness to changes in environment (scale A1). It was shown that nurses presenting a higher level of openness to changes taking place in the environment also had a higher level of readiness to ordain special foods (rho - Spearman’s correlation coefficient = 0.133), medical devices (rho = 0.229), potent drugs (rho = 0.208), narcotic drugs (rho = 0.155), psychotropic drugs (rho = 0.184), antibiotics, chemotherapeutic agents (rho = 0.159), and medicines previously ordered by a physician (rho = 0.113). Also, the level of self-evaluation of past educational development (C5 scale) increased the nurses’ readiness to ordinate all of the aforementioned drugs/agents. It is important, to note however, that the level of educational and professional goals alignment—employee and company (scale D2)—was less important when ordinating selected drugs/resources. Its influence was visible in the case of readiness to administer strong drugs (rho = 0.115), intoxicants (rho = 0.081), psychotropic drugs (rho = 0.081), antibiotics, chemotherapeutics (rho = 0.091), and medicines previously prescribed by a doctor (rho = 0.159).

The level of professional mobility awareness (B3 scale) significantly correlated only with the readiness to ordain two drugs/agents: medical devices (rho = 0.0129) and potent drugs (rho = 0.113). The level of effectiveness of on-the-job training (F6) had little effect on the ordination of medical devices (rho = 0.088) and psychotropic drugs (rho = −0.76), in the first case the correlation was positive and in the second negative. The impact of the level of professional mobility awareness (B3 scale) on the readiness to ordinate medicines/agents was not visible (Table 2).

It was shown that older nurses were less ready to administer strong drugs (rho = −0.158) than younger nurses. Younger nurses (rho = −0.100) had a higher level of readiness to ordain narcotic drugs. A higher level of preparation for the ordination of psychotropic drugs (rho = −0.118), and antibiotics and chemotherapeutics (rho = −0.130) was demonstrated by younger nurses. The age of the respondents significantly influenced the level of preparing the ordination of other drugs/agents (Table 3).

Our study showed that nurses work experience significantly influenced the willingness to ordain selected drugs/agents. It was found that with the increased length of service of nurses, willingness to ordain potent drugs decreased. It was also found that nurses with a work experience of 1–5 years had higher training to prescribe narcotic drugs (1.83 ± 1.11), psychotropic drugs (1.98 ± 1.19), antibiotics and chemotherapeutics (2.15 ± 1.18) than their colleagues with longer work experience (Table 4).

It was shown that the level of education of nurses significantly differentiated readiness to prescribe medicines/agents. In particular, nurses with Master’s degree and nursing specialization (3.95 ± 1.09) had a higher level of preparation for the prescribing of medical devices. Similarly, the highest level of readiness among this group of respondents concerned potent drugs (2.61 ± 1.20), narcotic drugs (1.90 ± 0.83), psychotropic drugs (2.02 ± 1.01) as well as antibiotics, and chemotherapeutics (2.07 ± 1.15) (Table 5).

Factors such as: marital status, experience of motherhood, place of work, place of residence, and form of employment did not significantly affect the readiness of the interviewed nurses to administer medicines and write prescriptions.

## 4. Discussion

The study is the first one in Poland to elaborate on the subject of nurses’ readiness to learn and develop in the context of new nursing rights regarding autonomous prescription of medication and continuation of the prescription for medication. Technological progress and the development in many fields of science enforce stronger and lasting relationships between education and employment [33]. New nursing put this group of professionals in need to engagement in personal professional development [1,2]. The analysis of the research results showed that for all the levels of readiness for learning and development average results prevailed and the highest level of readiness for learning and development was presented by nurses in terms of: openness to changes in environment, self-evaluation of past educational development, and effectiveness of on-the-job training [30,31,32].

Research on the readiness to learn and develop among employees qualified by the author of the RELD questionnaire, presented in the article, showed that the vast majority of results obtained by the respondents were on the level of average stents in all six subscales regarding general and professional readiness to learn and develop [30,31,32].

A factor that significantly influenced the readiness of nurses to learn and develop was the age and work experience of the respondents. Older people had a lower level of experienced professional mobility (*p* < 0.0001), effectiveness of educational and vocational goals achieved (*p* = 0.0002) and demand for vocational information (*p* < 0.0001), whereas people with work experience from 1 to 10 years had significantly higher scores on the professional mobility felt (*p* = 0.0007) than their longer working colleagues. This has been also confirmed by the results of A. Różański’s research showing the subscales relationship to learning and development and the length of service and the age of skilled workers [30]. In 2016, the average age of a nurse was 50.79 years, which is 6.6 years more than in 2008. Currently, the statistical Polish nurse is 52 years old, according to forecasts, it will be 60 in 2030. In addition, about 86% of people with the qualifications required to practice the nursing profession have reached the age of 42, and the largest professionally active group, as much as 17.77%, is in the range from 47 to 55 years. These are data that are of concern. According to a report by the Supreme Council, the Council of Nurses and Midwives is threatened by lack of substitution of generations, which means that more people will end their career than start it. Young people less and less often decide to take up the profession of a nurse or midwife, which is why it is referred to as the “demographically old professional group” [34]. A similar situation is occurring in other countries. In the US, the average age of nurses is 50, where the talk is about the upcoming “Silver Tsunami”, given that in about 10 years a significant part of the currently working nurses will retire [35,36].

Results presented by other researchers pointing to a natural decline in the ability to take up education among older people and a greater willingness of young people to develop in order to increase the possibility of getting a job or a better position [31,37]. This is confirmed by the results of a study conducted in the United States, in Arizona, regarding the nurse’s readiness to use different technologies during learning. Young people comprehensively use new methods of learning while older nurses find it difficult [38]. The results obtained seem to confirm the widespread belief that the interest in education is declining with the increase in seniority. However, in the current situation, where demographic changes and an aging population also affect the professional group of nurses, it will be extremely important to effectively motivate older employees to work and develop [34]. In a study conducted by McKenzie in Canada on the readiness of nurses to prescribe medication among patients with diabetes in primary care, the author analyzed the impact of the age and the education level of respondents on their readiness to prescribe medicines to their patients. The results of her research have shown a lack of connection between these variables and the readiness to prescribe medicines. Differences in the obtained research results may be due to the fact that the Canadian study was conducted on a much smaller group (less than 1/10th) of nurses surveyed than the research presented in this article: 70 vs. 756 [39].

Significantly higher results in terms of general readiness for learning and development were gained by nurses with at least undergraduate education and a specialization in nursing (A1—*p* < 0.0001; B3—*p* < 0.0001; C5—*p* < 0.0001. According to Andrew Palmer, the level of education is still a factor which determines the procurement of employment. The unemployment rate decreases with the level of education [33]. It is especially important in the case of healthcare professions and allows them to become professional and competent healthcare providers [40].

The research conducted showed that general and professional readiness for learning and development had a particular impact on the ordination of medical devices (OR = 1.79, 1.28–2.51, OR = 1.50, 1.11–2.02), potent drugs (OR = 2.90, 1.84–4.59), intoxicants (OR = 2.62, 1.10–6.24), psychotropic drugs (OR = 3.65; 1,99–6, 69, OR= 1.58, 1.12–2.21), antibiotics and chemotherapeutics (OR = 2.33, 1.21–4.45, OR = 1.83, 1.03–3,26), medications exclusively ordered by a physician (OR = 1.58, 1.18–2.10).

Another study at primary health care facilities regarding the nurses’ opinion on writing prescriptions showed that the vast majority pointed to medical devices, followed by medicines previously ordered by a doctor and foodstuffs for particular nutritional uses [41]. A similar position on the subject of prescribing medicines by nurses was expressed in another study by doctors [42].

In addition, the following factors that had a significant impact on the willingness of the nurses surveyed to ordain drugs/agents were: the level of openness to changes in the environment (scale A1), the level of perceived effectiveness in achieving educational and professional objectives (scale C5) and the level of perceived effectiveness of vocational training in the workplace. The factors also concern foodstuffs for particular nutritional uses and intoxicants.

The readiness of Polish nurses to learn and develop and raise their competencies is very important to expand the Polish list of drugs and the growing autonomy of Polish nurses in this respect as in the case of other countries. In Sweden, the list of medicines which can be prescribed by nurses to patients contains 230 kinds of drugs [5]. In the Netherlands, due to the large number of diabetic patients, nurses play an important role in providing care and issuing prescriptions for these patients [2,8]. Another model is used in Ireland, where Registered Nurse Prescribers are allowed to prescribe medicines, and selected psychotropic and intoxicating agents [2]. Nurse Prescribers in the United States have full autonomy in prescribing and this includes intoxicating and psychotropic medication [2,5,8]. In countries faced with problems of underfinanced health care and rapidly spreading diseases particularly HIV/AIDS (Zambia, Malawi, Ethiopia, Tanzania), nurses play an important role in prescribing antiretroviral drugs. In Uganda, due to the large numbers of patients with cancer and AIDS, nurses subscribe antiretroviral drugs and morphine to patients suffering from severe pain [2,26,27].

Due to the fact that prescription of medicines by nurses in Poland is a new competence, there are no studies assessing the degree of preparation of nurses to undertake these tasks.

In Poland, there is no current research and/or studies on the willingness of nurses to achieve different kinds of competencies, including those related to ordaining and prescribing drugs. This study is one of the first ones dealing with the importance and necessity to undertake research in the context of new nursing skills. It can therefore be assumed that nurses who are characterized by natural curiosity and the desire to learn new skills will take the challenge of completing the course without difficulty, which will allow them to write prescriptions.

## 5. Conclusions

The age and seniority of the nurses surveyed significantly affected the readiness to learn and develop and was negative. Nurses with higher education and additional qualifications had a higher readiness to prescribe medications and write prescriptions. Higher readiness for learning and development is matched by a greater readiness to take on new competencies in prescribing medicines. New competencies to prescribe medicines and write prescriptions extended to nurses in Poland create new opportunities for this professional group and are in line with changes in nursing in Europe and the rest of the world. The entitlements for nurses in Poland have a developmental character, which is exemplified by subsequent permissions, the so-called “nurse advice”, awarded to nurses in October 2019 [43]. New tasks can improve the prestige of the nursing profession and make it attractive to new generations and encourage many young people to choose nursing as their career path. It is very important to create behaviors conducive to learning willingness to improve their skills and readiness to undertake new tasks. A good way to check whether nurses are more willing to take up new permits would be to repeat the study after a period of a 5 years from the introduction of the law allowing nurses to write prescriptions.

There are potential limitations of the study that need to be taken into account while interpreting the results:

- Age of the respondents—the research was conducted in a group of nurses whose average age was 48 years, which is comparable with the age the majority of nurses working in Primary Health Care system in Poland.
- The short period of validity of the legal act extending the nurses’ rights to write prescriptions in relation to the start date of the tests.
- The research was carried out in the south-east of Poland and, in the future, other regions of the country should also be included in the study.

## Figures and Tables

**Table 1:** Level of nurses’ readiness for learning and development (RELD).

RELD		A1	B3	C5	D2	E4	F6
M		3.76	2.88	3.68	3.33	2.95	3.60
Mediana		3.75	3.00	3.71	3.33	3.00	3.50
SD		0.49	0.81	0.54	0.54	0.48	0.52
Minimum		2.17	1.00	1.86	1.67	1.33	1.25
Maximum		5.00	5.00	5.00	5.00	4.67	5.00
Percentiles	25	3.50	2.50	3.29	3.00	2.67	3.25
	50	3.75	3.00	3.71	3.33	3.00	3.50
	75	4.08	3.50	4.00	3.67	3.33	4.00
*p*		0.7212	0.9426	0.5256	0.2241	0.0761	0.5352

RELD—Readiness of Employees for Learning and Development; A1—level of openness to changes in environment; B3—level of professional mobility awareness; C5—self-evaluation of past educational development; D2—educational and professional goals alignment (employee and company); E4—inner need of an employee for the professional information; F6- effectiveness of on-the-job training; M—arithmetic mean; SD—standard deviation; *p*—level of significance.

**Table 2:** Readiness to ordain drugs/agents and the nurses’ readiness for learning and development.

Total (*N* = 756)		The RELD Questionnaire Subscales					
Types of Drugs/Foodstuffs		A1	B3	C5	D2	E4	F6
Foodstuffs for Particular Nutritional uses	rho	0.133	0.047	0.111	0.011	−0.010	0.033
	*p*	0.0002	0.1952	0.0023	0.7589	0.7824	0.3617
Medical Products	rho	0.229	0.090	0.177	0.005	−0.014	0.088
	*p*	<0.0001	0.0129	<0.0001	0.8957	0.6944	0.0161
Potent Drugs	rho	0.208	0.113	0.202	0.115	−0.032	−0.056
	*p*	<0.0001	0.0018	<0.0001	0.0015	0.3759	0.1261
Narcotic Drugs	rho	0.155	0.060	0.175	0.081	−0.053	−0.070
	*p*	<0.0001	0.1007	<0.0001	0.0258	0.1441	0.0541
Psychotropic Medicines	rho	0.184	0.055	0.190	0.081	−0.041	−0.076
	*p*	<0.0001	0.1331	<0.0001	0.0263	0.2637	0.0372
Antibiotics, Chemotherapy	rho	0.159	0.038	0.185	0.091	−0.028	−0.036
	*p*	<0.0001	0.2980	<0.0001	0.0125	0.4489	0.3241
Drugs Previously Ordered by a Doctor only	rho	0.113	−0.013	0.075	0.159	0.013	−0.005
	*p*	0.0018	0.7163	0.0399	<0.0001	0.7239	0.8831

RELD—Readiness of Employees for Learning and Development; A1—level of openness to changes in environment; B3—level of professional mobility awareness; C5—self-evaluation of past educational development; D2—educational and professional goals alignment (employee and company); E4—inner need of an employee for the professional information; F6—effectiveness of on-the-job training; rho—Spearman’s correlation coefficient; *p*—level of significance.

**Table 3:** Readiness to administer drugs/agents depending on the age of the respondents.

Types of Drugs/Foodstuffs		Total *N* = 756
Foodstuffs for Particular Nutritional uses	rho	0.021
	*p*	0.5672
Medical Products	rho	0.000
	*p*	0.9899
Potent Drugs	rho	−0.158
	*p*	<0.0001
Narcotic Drugs	rho	−0.100
	*p*	0.0059
Psychotropic Medicines	rho	−0.118
	*p*	0.0011
Antibiotics, Chemotherapy	rho	−0.130
	*p*	0.0003
Only Drugs Previously Ordered by a Doctor	rho	0.025
	*p*	0.4956

rho—Spearman’s correlation coefficient; *p*—level of significance.

**Table 4:** Readiness to ordain medicines/agents vs. work experience in the profession of nurse.

	Work Experience in the Profession of Nurse *N* = 756										
Drugs and Foodstuff Types	1–5 years		6–10 years		11–15 years		16–20 years		More than 20 years		*p*
	M	SD	M	SD	M	SD	M	SD	M	SD	
Foodstuffs for Particular Nutritional uses	2.62	1.19	2.59	1.12	2.83	1.41	2.64	1.37	2.61	1.38	0.8070
Medical Products	3.23	1.32	3.44	1.26	3.46	1.38	3.38	1.44	3.30	1.47	0.8166
Potent Drugs	2.43	1.21	2.41	1.10	2.21	1.32	1.94	1.16	1.87	1.09	<0.0001
Narcotic Drugs	1.83	1.11	1.50	0.74	1.75	1.04	1.57	0.88	1.42	0.71	0.0304
Psychotropic Medicines	1.98	1.19	1.59	0.80	1.92	1.11	1.76	1.09	1.58	0.92	0.0169
Antibiotics, Chemotherapeutics	2.15	1.18	1.88	1.03	2.04	1.16	1.70	1.02	1.63	0.98	0.0003
Only Drugs Previously Ordered by a Doctor	3.17	1.32	3.50	1.26	3.46	1.34	3.30	1.47	3.35	1.53	0.6887

**Table 5:** Readiness to ordain drugs/agents vs. education of respondents (*N* = 756).

Drugs or Foodstuffs Types	SNS		SNS + S		B.Sc.		B.Sc. + S		M.Sc.		M.Sc. + S		*p*
	M	SD	M	SD	M	SD	M	SD	M	SD	M	SD	
Foodstuffs for Particular Nutritional uses	2.61	1.35	2.66	1.44	2.57	1.30	2.42	1.33	2.69	1.37	3.05	1.38	0.3483
Medical Products	3.13	1.44	3.59	1.54	3.33	1.41	3.63	1.36	3.53	1.34	3.95	1.09	0.0005
Potent Drugs	1.81	1.06	1.95	1.23	1.97	1.16	2.05	1.09	2.44	1.21	2.61	1.20	<0.0001
Narcotic Drugs	1.45	0.77	1.51	0.84	1.48	0.81	1.63	0.94	1.61	0.87	1.90	0.83	0.0013
Psychotropic Medicines	1.56	0.89	1.70	1.05	1.66	1.02	1.89	1.29	1.82	1.03	2.02	1.01	0.0083
Antibiotics, Chemotherapeutics	1.59	0.92	1.82	1.19	1.63	0.95	1.97	1.20	2.13	1.16	2.07	1.15	<0.0001
Only Drugs Previously Ordered by a Doctor	3.28	1.51	3.44	1.49	3.42	1.50	3.55	1.41	3.43	1.31	3.22	1.35	0.6616

SNS—secondary nursing school; SNS + S—secondary nursing school and specialization in nursing, B.Sc—Bachelor of Science; B.Sc. + S—Bachelor of Science and specialization in nursing; M.Sc.—Master of Science; M.Sc. + S—Master of Science and specialization in nursing; M—arithmetic mean; SD– standard deviation; *p*—level of significance.

## Data Availability

The datasets generated and analyzed during the current study are available in the University of Rzeszow Repository (http://repozytorium.ur.edu.pl/handle/item/3693) as an Excel file under the name of the first author, and the title of the manuscript.

## Abbreviations

RELD	Readiness of Employees for Learning and Development
CPD	Continuum Professional Development
M	The arithmetic mean
SD	The statistical measure of the results spreading around the expected values
*p*	The level of significance was *p* < 0.005
